# A Flexible and Highly Sensitive Pressure Sensor Based on a PDMS Foam Coated with Graphene Nanoplatelets

**DOI:** 10.3390/s16122148

**Published:** 2016-12-16

**Authors:** Andrea Rinaldi, Alessio Tamburrano, Marco Fortunato, Maria Sabrina Sarto

**Affiliations:** 1 Nanotechnology Research Center Applied to Engineering (CNIS), Sapienza University of Rome, 00185 Rome, Italy; alessio.tamburrano@uniroma1.it (A.T.); marco.fortunato@uniroma1.it (M.F.); mariasabrina.sarto@uniroma1.it (M.S.S.); 2 Department of Astronautics, Electrical and Energy Engineering (DIAEE), Sapienza University of Rome, 00184 Rome, Italy

**Keywords:** pressure sensor, foam, graphene, nanoplatelets, PDMS, piezoresistivity

## Abstract

The demand for high performance multifunctional wearable devices is more and more pushing towards the development of novel low-cost, soft and flexible sensors with high sensitivity. In the present work, we describe the fabrication process and the properties of new polydimethylsiloxane (PDMS) foams loaded with multilayer graphene nanoplatelets (MLGs) for application as high sensitive piezoresistive pressure sensors. The effective DC conductivity of the produced foams is measured as a function of MLG loading. The piezoresistive response of the MLG-PDMS foam-based sensor at different strain rates is assessed through quasi-static pressure tests. The results of the experimental investigations demonstrated that sensor loaded with 0.96 wt.% of MLGs is characterized by a highly repeatable pressure-dependent conductance after a few stabilization cycles and it is suitable for detecting compressive stresses as low as 10 kPa, with a sensitivity of 0.23 kPa^−1^, corresponding to an applied pressure of 70 kPa. Moreover, it is estimated that the sensor is able to detect pressure variations of ~1 Pa. Therefore, the new graphene-PDMS composite foam is a lightweight cost-effective material, suitable for sensing applications in the subtle or low and medium pressure ranges.

## 1. Introduction

In the recent years, the development of smart systems and wearable health-care devices has drawn tremendous attention towards the development of new flexible highly-sensitive pressure sensors [1]. In particular, special interest has been focused on the study of innovative piezoresistive sensors characterized by high sensitive detection over a wide pressure range, easy signal processing, low cost and simple manufacturing process. 

A piezoresistive response was observed in elastomers loaded with conventional or nano-sized conducting fillers [2,3,4]. However, their manufacturing process is typically multistep and complex, since in general a homogeneous dispersion of fillers is obtained only through chemically modification with functional groups [5]. A relatively high loading is often necessary in order to achieve the target electrical conductivity, although it results in a critical degradation of the initial mechanical properties. Moreover, elastomer-based conducting nanocomposites are materials whose exploitation is complicated in those fields of applications requiring extremely lightweight and sensitive devices, able to detect pressures as low as 100 kPa [1]. Accordingly, elastomer-based conducting foams with an open-cells structure have been investigated recently in order to produce ultra-soft highly-performing piezoresistive pressure sensors, able to meet in the near future both the mechanical compliance and the electrical performance required for advanced health monitoring, medical diagnosis and electronic skin application. Basically, two different approaches are developed in the most recent literature. 

The first approach is founded on the pioneering work of Chen [6] and consists in realizing freestanding three-dimensional (3D) interconnected graphene networks through chemical-vapor deposition (CVD) of graphene on a sacrificial and etchable backbone of nickel. Because of their mechanical fragility [7], graphene foams are infiltrated with elastomers to increase the strength and flexibility. The material presented in [6] is characterized by a 20% variation in the electrical resistance when bended to a radius of 0.8 mm and by a 15% variation in response to a 50% uniaxial tensile strain.

The second approach is based on the fabrication of elastomeric foams internally coated with graphene or carbon nanotubes (CNT). In [8] the authors fabricated a conducting and soft material by dip coating of a commercial polyurethane (PU) foam with a thin layer of reduced graphene oxide (rGO). They demonstrated that the material can be used to detect pressure as low as 9 Pa with sensitivity of 0.26 kPa^−1^ for pressure up to 2.5 kPa and of 0.03 kPa^−1^ from 2.5 kPa to 10 kPa. Since in several engineering applications non-toxic, uninflammable and flexible materials are often required, others authors have investigated the potential use of polydimethylsiloxane (PDMS)-based porous templates to realize pressure sensors. In fact, PDMS is a particularly interesting material due to its low-cost, excellent elastic properties and biocompatibility. In [9], Han and co-workers obtained a piezoresistive pressure-sensitive sponge infiltrating a porous matrix of PDMS with functionalized single-walled CNTs. The CNT-PDMS sponge was characterized by compressibility up to 90% and by a non-linear monotonic decrease of the measured electrical resistance from the value of 10.6 MΩ at rest down to the value of 200 kΩ at maximum deformation. Based on [9], Amjadi et al. realized in [10] a sensitive footpad in which the integrated sensors show a linear electromechanical response with a sensitivity of ~10^−3^ kPa^−1^ for pressures up to ~480 kPa. 

In this paper, we investigate the piezoresistive performance of novel conducting PDMS foams coated with multi-layer graphene nanoplatelets (MLGs). At the best of our knowledge, there are no references and studies on this type of porous PDMS-graphene composite and its use as pressure sensor. MLGs are stacks of 2D graphene sheets, having thicknesses of a few nanometers and lateral dimensions up to ten microns. Recently, they have been used as nanofillers in polymer composites to produce highly sensitive piezoresistive strain sensors [11]. In fact, MLGs are particularly interesting as nanofillers, because they can be produced at low cost and in high quantities suitable for large-scale applications through the thermo-chemical exfoliation of intercalated graphite compound. However, the possibility of obtaining a MLG coating without binder on the surface of a porous skeleton of PDMS has not been reported yet. 

Therefore, the objective of this work is twofold. We describe a simple production method to obtain a highly-sensitive pressure sensor made with a MLG-coated PDMS foam. Moreover, we focus our attention on the piezoresistive properties of the coated foam under pressures up to 70 kPa. The stability and the sensitivity of the sensor response are also assessed. In particular, we demonstrate that the new sensor possesses a higher sensitivity compared to CNT-based sensors [9,10]. In addition, the effect of strain rate on both the mechanical and piezoresistive behaviors of the fabricated sensor is analysed.

As outlined in Section 2, porous PDMS is produced through a simple leaching process of sugar particles infiltrated with PDMS. Successively, the so-obtained PDMS foam is infiltrated with MLG/1-propanol colloidal suspension via drop casting, thus obtaining an electrical conductive porous soft material. Section 3 describes the different tests performed in order to characterize experimentally the produced samples; the results are presented and discussed in Section 4. In particular, optical and electron scanning microscopy (SEM) images of porogens and PDMS foams before and after infiltration are reported. The MLG morphology are also analyzed through atomic force microscopies (AFM). Next, the mechanical behavior of PDMS foams, obtained with two different cross-linking degrees, is investigated under compression at different quasi-static strain rates. Then, the electrical conductivity of the infiltrated foams is reported as a function of the MLGs weight fraction. Successively, we characterize the piezoresistive behavior of the produced MLG-PDMS foams with respect to a progressively increasing compression, discussing their performances as pressure sensors through the assessment of the sensitivity and stability of their response during quasi-static mechanical tests.

## 2. Materials and Methods

### 2.1. MLG-PDMS Production

**Figure 1:**
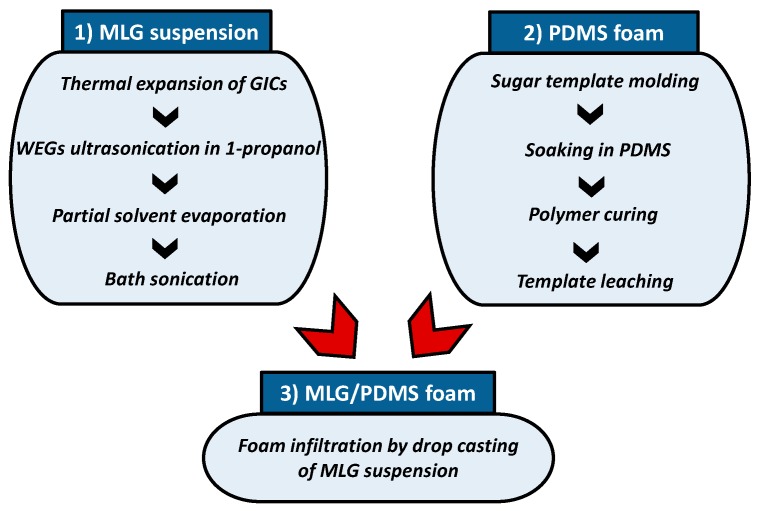
The main steps to obtain piezoresistive MLG/PDMS foams.

This section is aimed at describing the procedure developed for the production of soft porous MLG/PDMS materials with pressure sensing capabilities. As sketched in Figure 1, the process consists of three main distinct steps: (1) preparation of MLG-based colloidal suspensions; (2) production of PDMS open-cell foams; (3) infiltration of the porous PDMS with MLGs colloidal suspension.

#### 2.1.1. Colloidal Suspensions of MLGs

**Figure 2:**
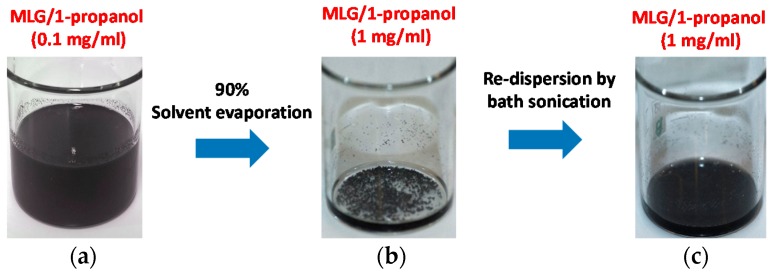
MLG suspension after: (**a**) ultrasonication, (**b**) solvent evaporation and (**c**) re-dispersion step.

MLGs are obtained applying the liquid-phase exfoliation process described elsewhere [12]. The used precursor is a commercially available Graphite Intercalation Compound (GIC) which is thermally expanded in a muffle furnace (in air) at 1150 °C for 5 s. The fast evaporation of the intercalated acid causes a volume expansion of the precursor material of more than a factor 200, leading to the formation of the so called Worm-like Expanded Graphite (WEG). Successively, 4.5 mg of WEG are dispersed in 45 mL of 1-propanol and exfoliated through ultrasonication, using a sonotrode tip operating with a symmetric duty cycle (1 s on-phase, 1 s off-phase). A thermo-cryostat is also used to maintain the suspension at 10 °C, thus limiting solvent evaporation and overheating of the probe tip. The used solvent prevents the polymer from swelling ([13]) during the subsequent infiltration process step, because it is a volatile alcohol with a low boiling point (97 °C) and low solubility in PDMS. An initial MLG concentration of 0.1 mg/mL is used in order to guarantee an optimum exfoliation of WEGs in 1-propanol and to obtain a stable colloidal suspensions of MLGs as discussed in [14]. The as-obtained MLG/1-propanol suspension (Figure 2a) is then boiled in order to evaporate 90% of the solvent, obtaining a ten times higher concentration of MLGs, which agglomerate in large clusters (Figure 2b). Finally, the nanofiller agglomerates are re-dispersed through the use of a bath sonicator for a few minutes (Figure 2c). The resulting colloidal suspension is stable, and it shows a negligible filler precipitation for more than 30 min, which is the time necessary to complete the subsequent infiltration step.

#### 2.1.2. PDMS Foam Production

**Figure 3:**
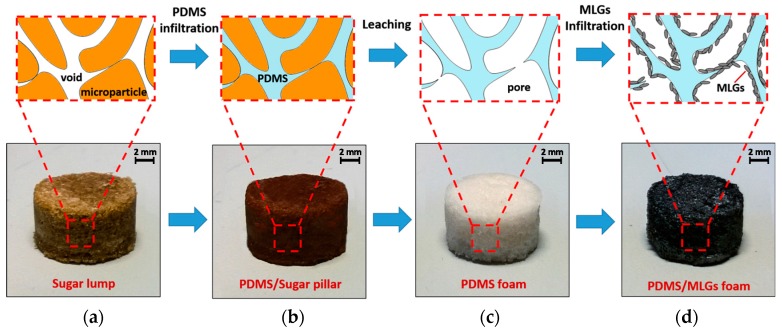
Products at the end of the distinct production steps: (**a**) sugar lump extracted from molds; (**b**) sample after PDMS infiltration; (**c**) PDMS foam after leaching; (**d**) PDMS foam after MLGs infiltration.

The production of PDMS foams is performed following the direct template method, which consists in the replication of the inverse structure of a preformed leachable template. Commonly, commercially available sugar lumps or sugar/salt particles are used to form sacrificial well-connected templates [15,16,17,18,19]. In the present work templates are obtained by compacting dark brown sugar particles having an average size of 270 μm (see Section 3.2). In particular, sugar particles are firstly filtered with a sieve (mesh size of 700 μm) to remove agglomerates and then compressed in a cylindrical metallic mold at a constant pressure of 250 kPa for 1 min. Liquid PDMS is prepared by mixing and stirring for 5 min the prepolymer (Sylgard 184 Part A) and the curing agent (Sylgard 184 Part B) in different weight ratios in order to produce material families with different elastic properties [20,21]. Subsequently, the sugar templates (Figure 3a) are extracted from the molds, dipped in PDMS and placed inside a chamber overnight at −70 kPa to ease the air bubbles removal and the infiltration of interstices via capillary forces. Successively, PDMS-soaked templates are cured in an oven for 10 min at 150 °C. Then, after the removal of the exceeding PDMS from the faces of the PDMS/sugar pillars, the samples (Figure 3b) are put in demineralized water inside an ultrasonic cleaner for 120 min. Hence, sugar porogens are leached out, so than a 3D highly-interconnected PDMS foam is obtained (Figure 3c). The final sample has a cylindrical shape, with a nominal diameter *D* of about 11.5 mm and thickness $h_{0}$ of 6.5 mm. The porosity Φ of foams is determined with [22]:

(1) $$\Phi =1-\rho_{foam}\cdot {\rho_{PDMS}}^{-1}$$

where $\rho_{PDMS}$ and $\rho_{foam}$ are the density of PDMS and the apparent density of the porous PDMS, respectively.

#### 2.1.3. PDMS Foam Infiltration

The produced PDMS foams are infiltrated via drop casting with the MLG/1-propanol suspensions, thus realizing electrically conductive porous polymer samples. The adopted method is similar to the one reported in [19], which describes the fabrication of a porous material with superhydrophobic and oleophilic properties. The porous material is obtained through the syringe-injection of a graphene-DMF solution in a PDMS sponge. In the present paper the infiltration is performed in a small environmental chamber at fixed temperature of 110 °C to facilitate the solvent evaporation. The process consists of several cycles, each one involving the infiltration of 0.25 mL of suspension with a Pasteur pipette. After evaporation of the solvent, MLGs remain trapped inside the pores, and realize a thin conducting coating adhering through interfacial forces (Figure 3d). The MLG weight concentration (*θ* wt.%) with respect PDMS is obtained from the measurement of the sample weight, before and after infiltration. The produced MLG-PDMS foam samples are lightweight, soft and easily squeezable.

### 2.2. Experimental Tests

#### 2.2.1. Morphological Characterizations

Morphological analyses are carried out on the porogens, on the produced nanostructures and on the foams. The porogens lateral dimension are investigated using an Eclipse LV150 optical microscope (Nikon, Tokio, Japan) equipped with an Axiocam ERc5 camera (Carl Zeiss, Oberkochen, Germany) and a 10× lens. The thickness and the lateral dimensions of MLGs are investigated by atomic force microscopy (AFM) using a Dimension Icon AFM (Bruker-Veeco, Billerica, MA, USA) operated in tapping mode. Samples for AFM tests are produced spraying the colloidal suspensions of MLG/1-propanol over a heated Si wafers coated by 300 nm of SiO_2_. The foams morphology, before and after MLG infiltration is characterized using a Zeiss Auriga Field Emission Scanning Electron Microscope (FE-SEM, Carl Zeiss, Oberkochen, Germany).

#### 2.2.2. Mechanical, Electrical and Electromechanical Characterizations

Mechanical tests of the produced PDMS-based cylindrical specimens (diameter *D* = 11.5 mm, thickness $h_{0}$ = 6.5 mm) are performed at room temperature using a universal testing machine (Instron 3366, ITW Test and Measurement Italia S.r.l. – Instron CEAST Division, Pianezza, Italy), equipped with a 10 N load cell and custom-made parallel-plate compression fixture (Figure 12). The barreling effect due to friction in the unconfined compression tests was minimized by lubricating the parallel surfaces of the PDMS cylinders. Moreover, the presence of toe region at the beginning of the stress-strain curve has been taken into consideration and compensated after the test.

The electrical resistance of the foams after MLG infiltration is measured using a two-wire volt-amperometric technique, through a Keithley 6221 dc/ac current source (Keithley, Cleveland, OH, USA) connected to a Keithley 2182 nano-voltmeter (Keithley, Cleveland, OH, USA) and controlled by a laptop for data acquisition. In particular, the foams are firstly sandwiched between two aluminum platens, working as electrodes. Notice that the platens have been properly designed to be screwed to the compression fixture. The wires are then attached to the electrodes and connected to the measuring units. The effective dc conductivity (*γ_eff_*) of each MLG/PDMS foam sample is obtained from the corresponding measured resistance, diameter and thickness values.

Moreover, in order to investigate the piezoresistive response and performances of the fabricated materials as pressure sensors, quasi-static uniaxial compression tests are performed in conjunction with resistance measurements using the previously described instrumentation. 

## 3. Results and Discussion

### 3.1. Produced Samples

We produced two different PDMS foam sets, namely F10 and F20. The former type is obtained by infiltrating the leachable templates with a stoichiometric ratio 10:1 of PDMS prepolymer and curing agent, whereas the second sample set is produced with twice the amount of Sylgard 184 Part A (20:1). The average physical properties of the produced foams are summarized in Table 1.

The PDMS density ($\rho_{PDMS}$), obtained as reference from the measured weight and volume of bulk PDMS samples, is nearly constant under various mixing ratios [23]. In fact, the values reported in Table 1 are comparable to the ones declared in the material data sheet (1030 kg/m^3^) [20]. The apparent density of both foam types, evaluated as the mass of the polymer 3D skeleton divided by the total volume including porosity, is approximately three times lower than the one of bulk PDMS. Consequently, the porosity calculated using Equation (1) results to be around 65%.

The different base/curing agent mass ratio affects the cross-linking degree of PDMS and consequently its elastic properties [20,21]. In fact, as shown in Table 1, the Young’s modulus of PDMS (*E_PDMS_*) is reduced of a factor ~3.63 by halving the amount of curing agent. As a result, foams F20 are softer than the ones of the F10 family, and this is clearly revealed by the values of their elastic moduli (*E_foam_*) reported in the same table. The values of *E_PDMS_* and *E_foam_* are evaluated from the initial slope of the stress-strain response of material samples measured through uni-axial compression tests (see Section 3.3). 

The produced PDMS foams have been then used to produce soft pressure sensors. In particular, five different specimens of MLG/PDMS foams are obtained as described in Section 2.1.3 using the F10 family (MLG-F10) by drop casting different amount (i.e., 0.75, 1, 1.5, 2, 3 and 4.5 mL) of MLG colloidal suspension. In a similar way, other five infiltrated foams are obtained with samples of the F20 family (MLG-F20). The weight percentage *θ* [wt.%] of MLGs in the final infiltrated material with respect to the PDMS weight is reported in Table 2. 

### 3.2. Morphological Analyses

**Figure 4:**
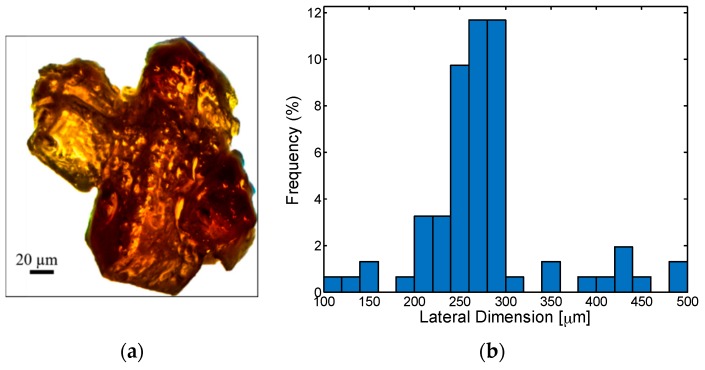
Optical image of a porogen (**a**); and porogen size distribution (**b**).

**Figure 5:**
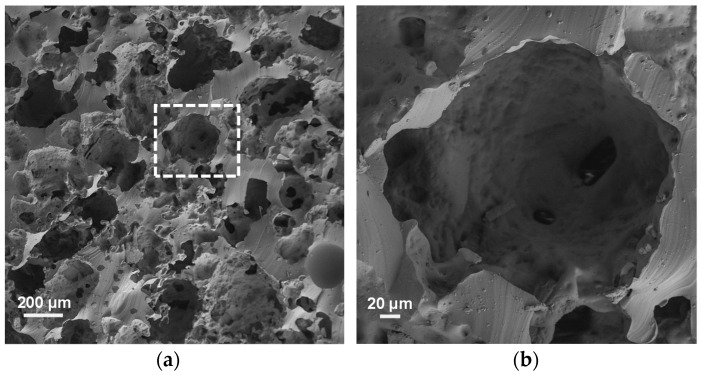
(**a**) SEM image of the cross-section of a PDMS foam after leaching of a preformed sugar based template; (**b**) Higher magnification view of the pore marked in (**a**).

The mechanical behavior of the polymeric foams is related to their microstructure and to the properties of the polymer itself. Their physical characteristics, such as pore dimensions and porosity, depend on the shape, size and amount of the porogens. The degree of inter-connectivity between pores is affected by the fusion process between adjacent particles of the template prior to the polymer infiltration. In Figure 4a, we show the optical image of fused sugar crystals. Through the analysis of hundreds of pictures, the frequency distribution of the maximum lateral dimension of porogens is extracted and plotted in Figure 4b. It can be noted that most of the particles are characterized by a maximum size of 250–300 μm, with an average value of nearly 270 μm. Figure 5 shows the cross section of a PDMS foam after leaching. As expected, the polymer structure displays the negative replica of the sugar particles based template: pores have dimensions of few hundreds of micrometer and are composed of microperforated walls, leading to an open-cell porous structure. The pore interconnectivity is fundamental for the final realization of a soft conductive foam. In fact, a closed cell sponge would be denser and, most importantly would impede the infiltration of the MLG/propanol suspension throughout the material. It is important to remark that the size of nanoplatelets in 1-propanol also represents a critical parameter as it affects the liquid flow through the foam and the electrical properties of the porous structure. The proper setting of the process parameters during the thermal expansion of GICs and WEG sonication allows to optimize MLG exfoliation [14], thus avoiding to plug foam pores with too large nanoplatelets or to produce pulverized graphene flakes with very low-aspect ratio.

**Figure 6:**
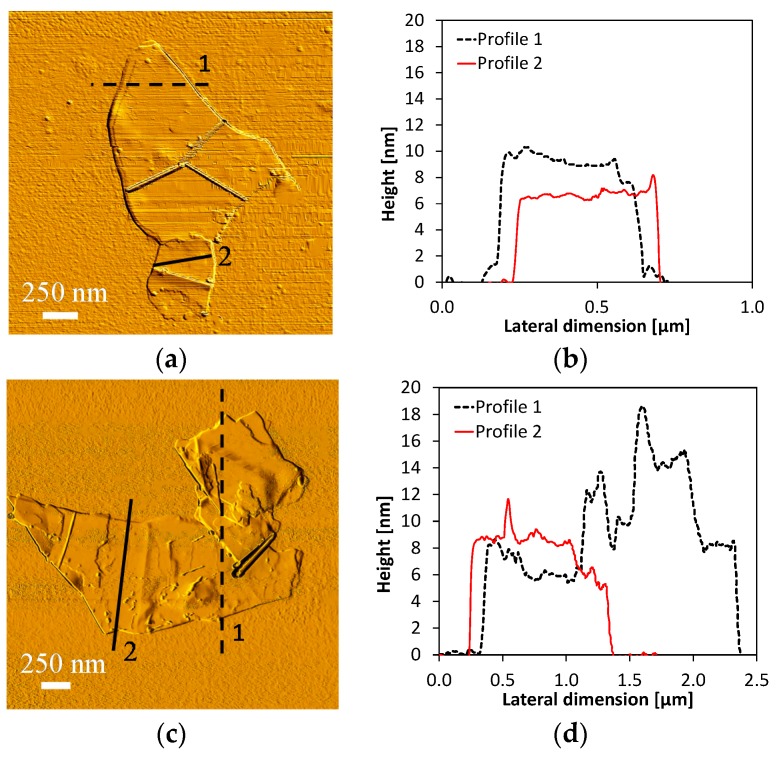
AFM images of a MLG flake and corresponding height profiles, before (**a**,**b**) and after (**c**,**d**) solvent evaporation and re-dispersion.

Figure 6a,c shows, as an example, two AFM images of MLGs with their measured thickness profiles (Figure 6b,d). An extensive investigation demonstrated that the produced multilayer graphene sheets have an average thickness of 8–10 nm and lateral dimensions in the range of few micrometers.

**Figure 7:**
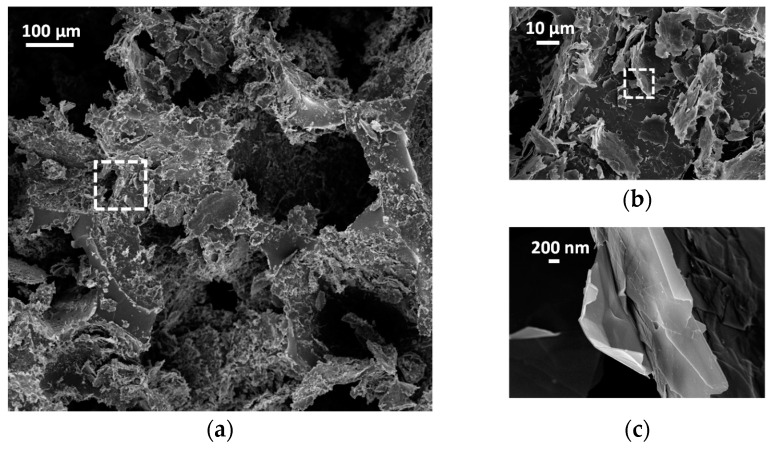
(**a**) SEM image of a MLGs coated PDMS foam; (**b**) Higher magnification view of the area marked in (**a**); (**c**) Higher magnification of flakes marked in (**b**).

After the drop casting process, MLGs adhere to the cell walls of the foam, realizing a coating of the PDMS backbone and mimicking its three dimensional structure. This is clearly observed in the SEM images of Figure 7.

### 3.3. Mechanical Characterizations

**Figure 8:**
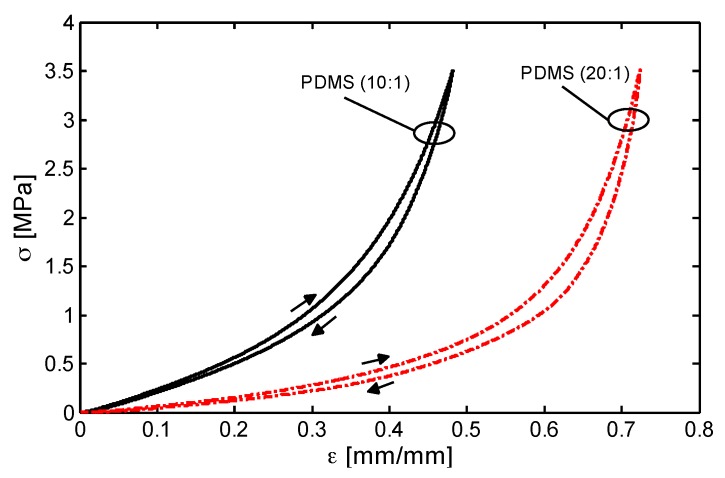
Stress-strain response of two bulk PDMS samples produced with a stoichiometric ratio 10:1 and 20:1 of PDMS prepolymer and curing agent for a loading-unloading compression test.

At first, cylindrically-shaped bulk PDMS samples obtained with two different base/curing agent mass ratio (i.e., 10:1 and 20:1) are subjected to a uniaxial quasi-static compression test at a crosshead speed of 1 mm/min up to a maximum stress of 3.5 MPa. The loading/unloading stress (*σ*)—strain (*ε*) responses are showed in Figure 8. The presence of small hysteresis loops (area between loading and unloading curves) are related to the viscoelastic properties of the material [24]. The curves demonstrate distinctly the superior flexibility of the PDMS specimens produced with the lowest content of curing agent (i.e., 20:1) in the whole investigated deformation range. This is also confirmed by the values of the compressive Young’s modulus *E_PDMS_*, evaluated at low strain in the linear region and reported in Table 1. 

**Figure 9:**
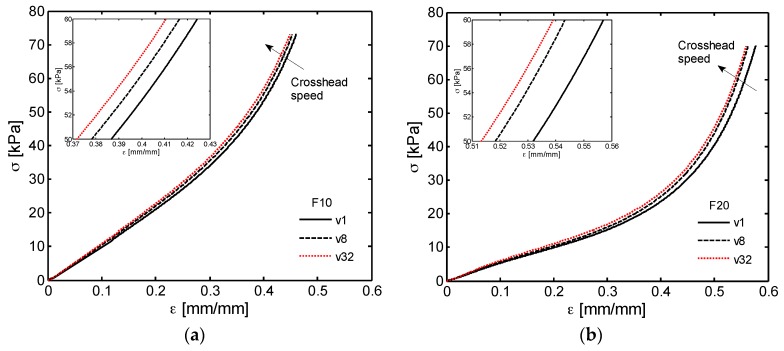
Strain rate dependent stress-strain characteristics of the (**a**) F10; and (**b**) F20 foams (*v*_1_ = 1 mm/min, *v*_8_ = 8 mm/min, *v*_32_ = 32 mm/min).

Monotonic compression tests are then performed to investigate the response of PDMS foams belonging to both F10 and F20 families. The obtained stress-strain characteristics are plotted in Figure 9a,b up to 70 kPa. Due to the lower cross-linking degree of the PDMS used, foam of the F20 family shows higher deformations than samples of the F10 one at the same stress level. Notice that the characterization is performed at different crosshead speeds *v*, ranging from *v*_1_ = 1 mm/min to *v*_32_ = 32 mm/min, which correspond to a quasi-static strain rate ($\dot{\varepsilon }$) of ~0.0026 s^−1^ and ~0.082 s^−1^, respectively [25]. The reason is that polymeric foams generally exhibit high degree of sensitivity to $\dot{\varepsilon }$ [26], which is defined as the derivative of the strain with respect to time or, equally, as the ratio between the crosshead speed *v* and the original thickness *h*_0_ of the gradually compressed sample. Such dependency of the stress-strain curve from the cross-head speed is usually related to the properties of PDMS and to the presence of air inside the foam. In our experiment, as shown in Figure 9, we observed a very slight change of the strain-stress response for increasing values of the crosshead speed: stepping up the crosshead speed from *v*_1_ to *v*_32_ the stiffness and compressive strength increase to a small extent, especially at higher induced strains. Tests conducted at much higher strain rates would be of special interest for impact and shock absorbing applications [25], but are out of the scope of the present work.

A similar trend was observed in [27] for PDMS scaffolds with porosity and pore size similar to the ones of the materials produced in this study, but fabricated through vacuum-assisted resin transfer molding and leaching of NaCl porogens instead of sugar particles.

It is interesting to note that, typically the compressive deformation of polymeric foams exhibits three distinct regimes. At small strain, the behavior is approximately linear elastic due to the cell wall bending and the curve slope is equal to the Young’s modulus of the foam *E_foam_*. The second region, known as plateau, is characterized by a progressive deformation at relatively constant stress due to elastic buckling, plastic yielding or crushing of cell walls. The width of plateau generally depends on the pore size and the thickness of the sample. During foam cell collapsing, the plateau region can also manifest a positive slope gradually increasing with strain related to the irregularities of cell geometry and the progressive hardening of the polymeric material. In the third regime, due to densification, the foam begins to behave like a compacted solid material and the stress increases more rapidly with strain [25,26,28,29].

**Figure 10:**
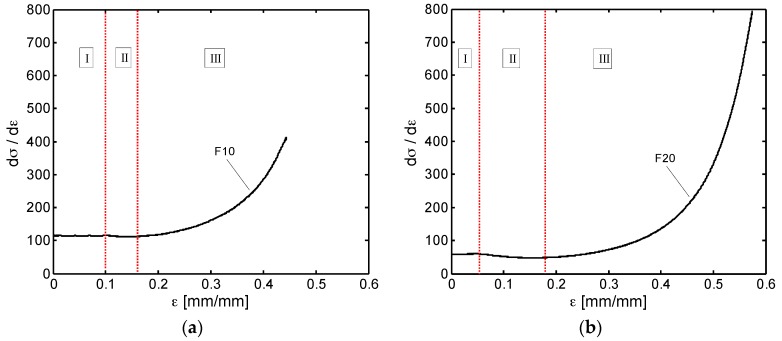
First stress derivative of a F10 (**a**); and F20 (**b**) foams pointing out the three foam transition regimes.

The above mentioned three characteristic phases of deformation are not well defined in Figure 9 mainly because of the random size and shape distribution of the pores in the produced PDMS foams. In order to highlight the transitions from one regime to the other for both the foam types (i.e., F10 and F20 families), we report in Figure 10 the stress first derivative with respect to strain (d*σ*/d*ε*).

The initial horizontal portion of the curves (Zone I) identifies the strain range for the linear elastic response of F10 and F20 foams, that is Young’s moduli *E_foam_* reported in Table 1. It can also be noted, that the transition from the linear elastic region to the plateau one (Zone II) occurs approximately at strain of 0.1 mm/mm and 0.05 mm/mm in Figure 10a,b, whereas the densification regime (Zone III) presumably begins at strains higher than 0.15 mm/mm and 0.18 mm/mm for the F10 and F20 foams, respectively.

### 3.4. Electrical Characterization

**Figure 11:**
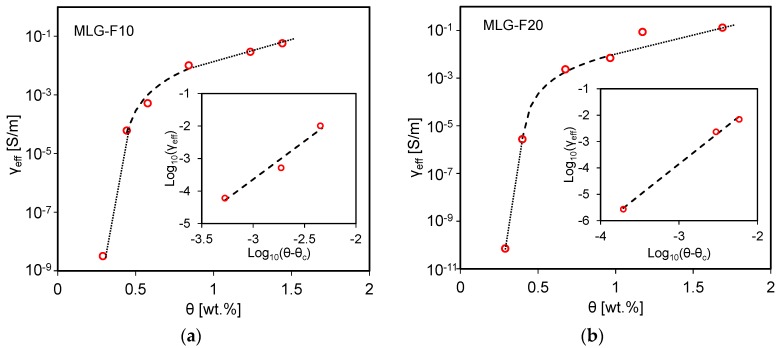
Effective DC conductivity of samples of the (**a**) MLG-F10; and (**b**) MLG-F20 foam types, for different weight concentration of MLGs.

The effective dc electrical conductivity (*γ_eff_*) of the coated foams MLG-F10 and MLG-F20 as function of the MLGs weight fraction *θ* [wt.%] is reported in Figure 11a,b respectively. As expected, *γ_eff_* increases with *θ*. In particular, between the 0.3 wt.% and 0.4 wt.% of MLGs, *γ_eff_* increases steeply of about four orders of magnitude for both the foam typologies, whereas a much slower variation is observed at concentration higher than 1.2 wt.%. 

Notice that this trend resembles the common electrical response change of a composite constituted by a dielectric matrix loaded with different concentrations of conducting nanoparticles [11]. For this reason, the measured data in Figure 11a,b are fitted using the well-known power law equation generally describing the insulating-conducting transition of a two-phase composite filler system, due to percolation:

(2) $$\begin{array}{ccc} \gamma_{eff}\propto {\left(\theta -\theta_{c}\right)}^{t} & \text{for} & \theta >\theta_{c} \end{array}$$

being *θ_c_* the percolation threshold and *t* the critical exponent. The straight lines in the insets of Figure 11 represent the least-squares fit of log_10_(*γ_eff_*) versus log_10_(*θ* − *θ_c_*) [11]. The fitting curve parameters are then reported in Table 3.

The comparison between the two fitting curves and the corresponding data in Table 3 demonstrates the almost equivalent dc electrical behavior of the two types of infiltrated foams. In fact, *γ_eff_* depends primarily on the characteristics of the filler, on the amount of infiltrated MLGs and on the porosity of the foams, which are very similar in all produced samples of both F10 and F20 families. 

### 3.5. Electromechanical Characterizations of MLG/PDMS Foams

**Figure 12:**
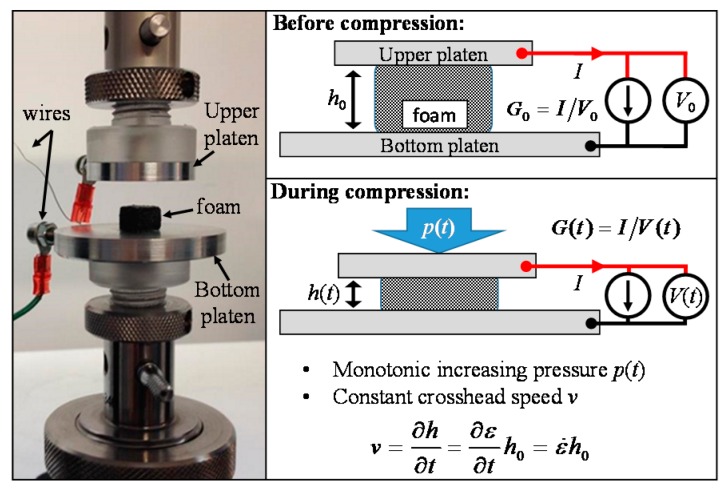
Adopted parallel compression platen system for the pressure tests and corresponding sketch of the electromechanical measurements.

The cross-linker concentrations of PDMS is a fundamental parameter affecting the mechanical properties of the foams, as discussed in Section 3.3. Only the MLG-F20 samples, due to their superior elasticity and softness with respect to the ones of the F10 typology have been then considered for the subsequent tests aimed at assessing their piezoresistive behavior. The feasibility to exploit the produced coated foams as piezoresistive pressure sensors has been investigated by measuring their electrical conductance *G* in μS, when they are subjected to a gradually increasing uniaxial compressive load as sketched in Figure 12. In particular, we performed the electromechanical tests on the specimens of Table 2 infiltrated with a MLG concentration of 0.96 wt.%, because this type of foam presents a value of effective conductivity *γ_eff_* just beyond the knee of the percolation curve of Figure 11b. This guarantees that resistance of the tested sample is much lower than the input resistance of used measurement equipment (i.e., ~10 GΩ). Samples containing a higher concentration of MLGs are not taken into consideration for the electromechanical tests because their sensitivity as pressure sensor to an applied stress would be much lower, as already demonstrated in case of piezoresistive nanocomposites [11].

**Figure 13:**
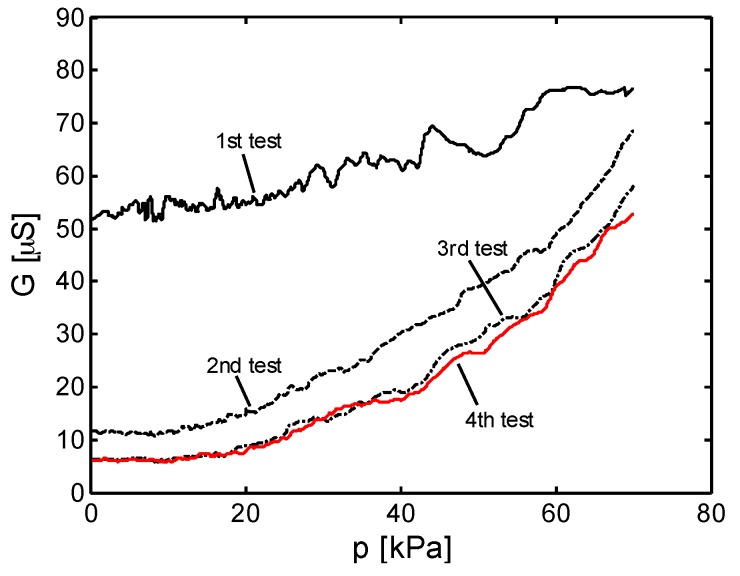
Mechanical stabilization of a MLG-F20 foam based pressure sensor: conductance *G* measured as function of applied pressure *p* during consecutive quasi-static monotonic loading tests.

The black solid line in Figure 13 shows the variation of *G* for the MLG-F20 specimen as a function of the applied pressure *p* (or stress *σ*) in kPa during the first test performed with a crosshead speed *v*_1_ = 1 mm/min. The electromechanical response repeatability of the fabricated sensors has been investigated performing consecutive quasi static monotonic compression tests. Notice that, despite the significant decrease of the conductance in Figure 13 after the first and second test, the piezoresistive signal stabilizes rapidly. A similar behavior has been already observed in nanocomposite-based strain sensors due to the occurrence of damages at micro- and nanoscale and inter-filler separation [11,30,31,32]. Likewise, in the case of infiltrated foams, the response variations can be attributed to the irreversible transformations of the tridimensional MLG percolation network coating the cell walls, especially during the first applied mechanical loadings. 

**Figure 14:**
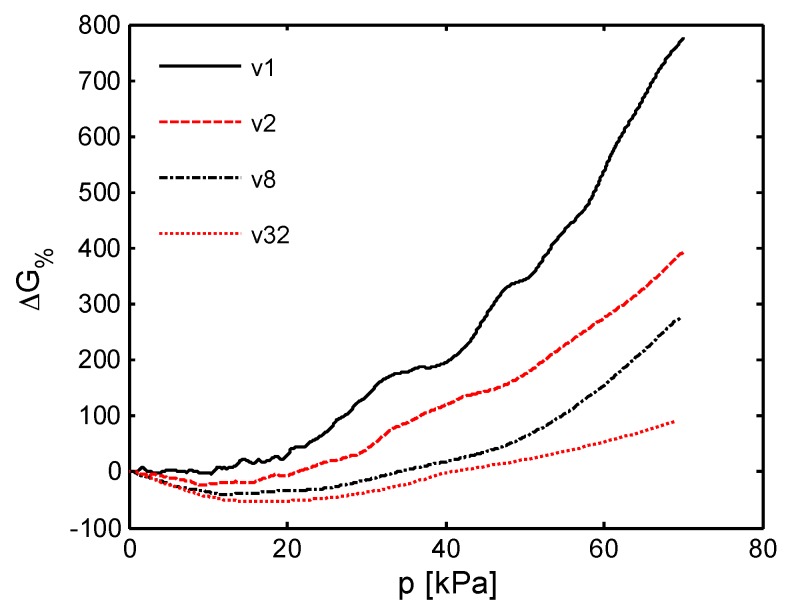
Relative percentage conductance variation as function of the applied pressure *p* at different crosshead speed (*v*_1_ = 1 mm/min, *v*_2_ = 2 mm/min, *v*_8_ = 8 mm/min, *v*_32_ = 32 mm/min).

**Figure 15:**
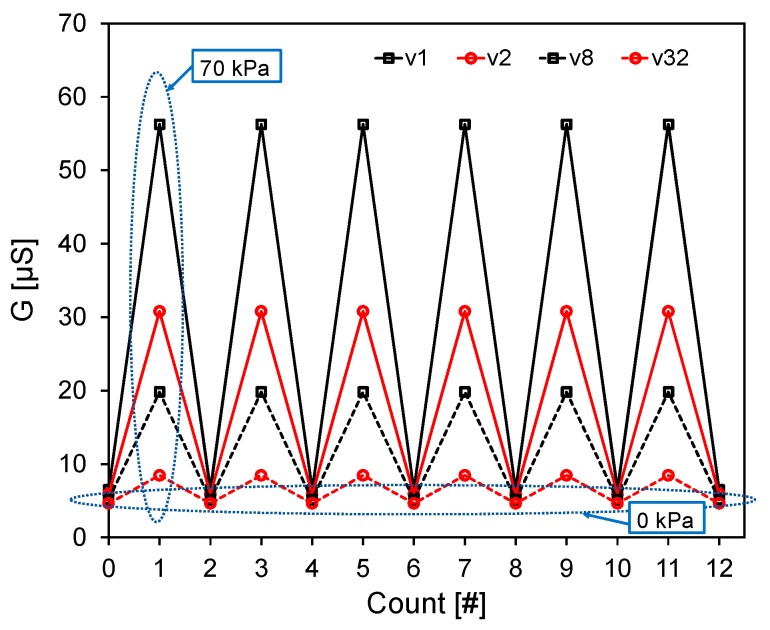
Conductance measured at the beginning (0 kPa) and at the end (70 kPa) of six consecutive monotonic loading tests repeated at different crosshead speeds.

**Figure 16:**
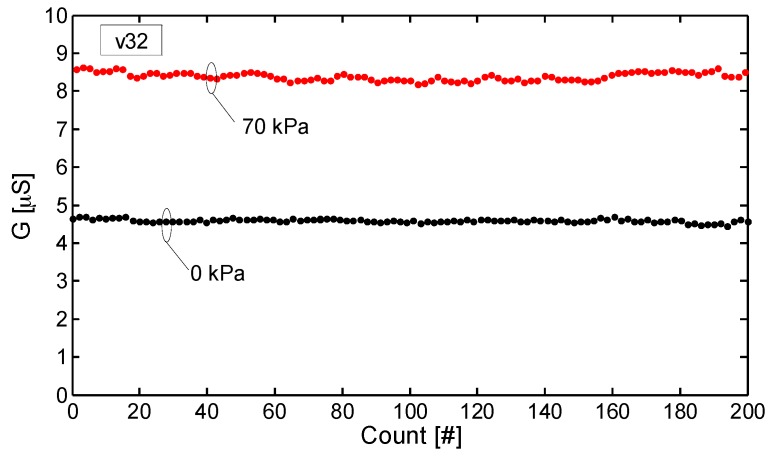
Conductance measured at the beginning (0 kPa) and at the end (70 kPa) of one hundred consecutive monotonic loading tests carried out at the crosshead speed *v*_32_.

The response of the sensor is then investigated at different crosshead speeds (*v*_1_ = 1 mm/min, *v*_2_ = 2 mm/min, *v*_8_ = 8 mm/min, *v*_32_ = 32 mm/min). In particular, Figure 14 shows the relative percentage electrical conductance variation as a function of the applied pressure in kPa:

(3) $$\Delta G_{\%}=100\frac{\Delta G}{G_{0}}=100\frac{G(p)-G_{0}}{G_{0}}$$

where *G*_0_ is the conductance in the unloaded condition and *G*(*p*) is the measured conductance with an applied pressure *p* (i.e., stress *σ*). Differently from the mechanical properties, the sensor piezoresistive behaviour is affected sensibly by the strain rate. At the lowest speed *v*_1_ (i.e., $\dot{\varepsilon }$ ~ 0.0026 s^−1^), Δ*G*_%_ is almost constant up to a pressure limit *p*_lim_ ≈ 10 kPa. For higher pressures (i.e., *p* > *p*_lim_), when corresponding to the densification regime of the foam, Δ*G*_%_ shows a monotonic increase. Differently, at higher cross-speed velocities (i.e., *v*_2_, *v*_8_ and *v*_32_), the sensor shows a negative and almost linearly decreasing Δ*G*_%_ up to *p* ≈ *p*_lim_. Beyond that value Δ*G*_%_ reverses the trend and starts to increase with a rising rate which is higher for lower speeds. Although the piezoresistive sensor output undergoes significant changes in response to different velocities, it shows a high repeatability after the mechanical stabilization shown in Figure 13. This fact is confirmed by the results reported in Figure 15 and Figure 16. Figure 15 displays the conductance recorded at the beginning and at the end of each of the six monotonic loading tests performed in sequence (with an applied pressure ranging from 0 kPa up to 70 kPa), and repeated for the different crosshead speeds *v*_1_, *v*_2_, *v*_8_ and *v*_32_. Figure 16 shows the conductance values measured at two different applied pressures during one hundred consecutive compression tests carried out at the maximum speed *v*_32_ (the most severe test condition). It is noted that the sensor provides a high repeatable response throughout all the cycles. In particular, with reference to Figure 16 it is observed that the conductance average values at 0 kPa and 70 kPa of applied pressure are 4.65 μS and 8.45 μS, respectively with a standard deviation of 0.15 μS and of 0.16 μS.

**Figure 17:**
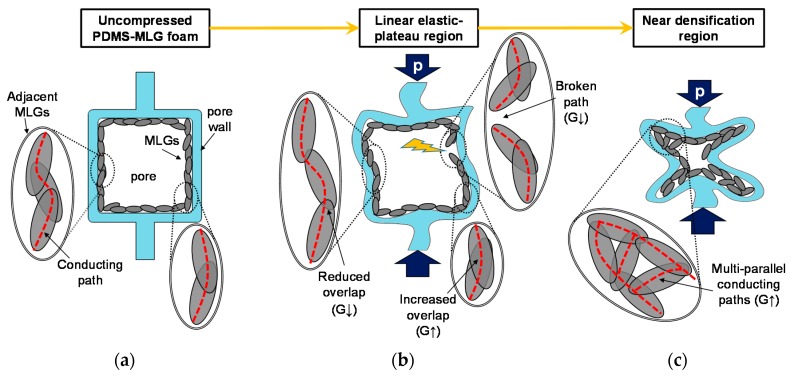
MLG coating modification during compression and its effect on the overall conductance *G* of the foam. (**a**) Before loading, MLGs adhere to the PDMS foam walls and create the conducting paths that affect the value of the foam initial conductance *G*_0_; (**b**) Applying a pressure in the linear-plateau region of the foam response, the MLG coating undergoes modification due to the sliding between flakes and due to the conducting path degradation occurring especially at higher crosshead speeds; (**c**) In proximity of the densification region of the foam, at higher pressures, the collapsing cells cause the formation of additional parallel conducting paths and the increase of *G*.

It is believed that the mechanism responsible for the variation of the conductance at rest of the sensor from its initial value *G*_0_ is attributed to the rearrangement of the conducting network within the MLG-coating deposited over the pore walls of the foam, induced by the applied strain-stress. In fact, the deformation of the polymeric backbone can affect the MLGs inter-distances, causing a variation of the contacts as well as the tunneling phenomena between neighboring sheets. Such mechanism is sketched in Figure 17.

Without an applied pressure (Figure 17a), the MLGs-coating adheres and mimics the PDMS foam inner structure, as also seen in the SEM images. At the lowest investigated strain rate, in the linear elastic and plateau regions, *G* is practically constant. This means that the overall electrical resistance of conductive paths created by the MLGs remains almost unchanged. In fact, during loading, some parts of the foam walls are subjected to compressive stress while others to stretching. Therefore, adjacent MLGs adhering to the PDMS skeleton surface are induced either to increase or decrease their overlapping area (if in contact, otherwise reduce or increase their mutual distances) (Figure 17b). We can speculate that this behavior is possible thanks to the MLGs good adhesion with the foam and to the low friction between sliding flakes [33]. As a result, although the MLGs relocation leads to modify the value of the local contact resistances, from a statistical point of view the overall effective resistance of the foam remains almost constant.

At higher speeds the coating is not able to comply with substrate deformation; consequently, MLGs tend to detach from PDMS provoking the deterioration of same conducting paths (Figure 17b). This phenomenon induces a positive piezoresistivity [34], consisting in an increment of the foam resistance (i.e., *G* and Δ*G*_%_ decrease) with an increasing pressure. On the contrary, in proximity of the densification region, the cell walls collapse and generate additional electrical contacts between MLGs, creating multiple parallel conducting paths (Figure 17c). Therefore, the foam manifests a negative piezoresistivity, consisting in an increasing of *G* as the applied pressure becomes more intense.

**Figure 18:**
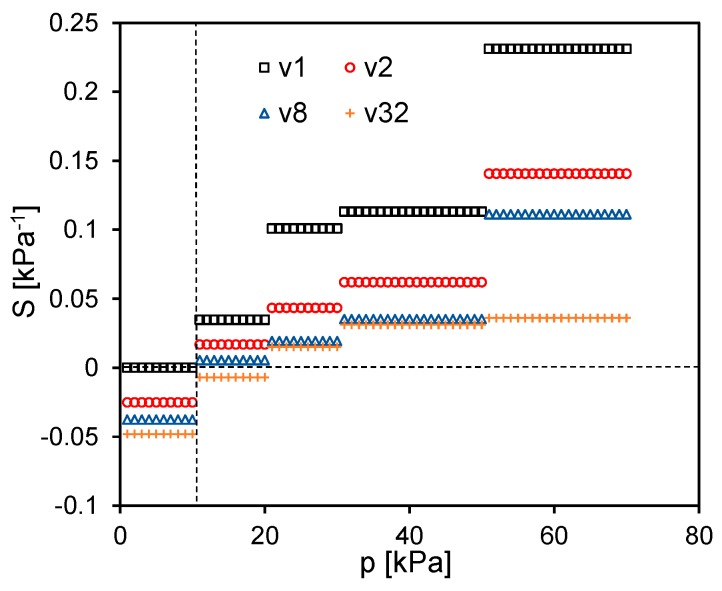
Sensitivity ranges of the fabricated sensor as function of the applied pressure and strain rate.

**Figure 19:**
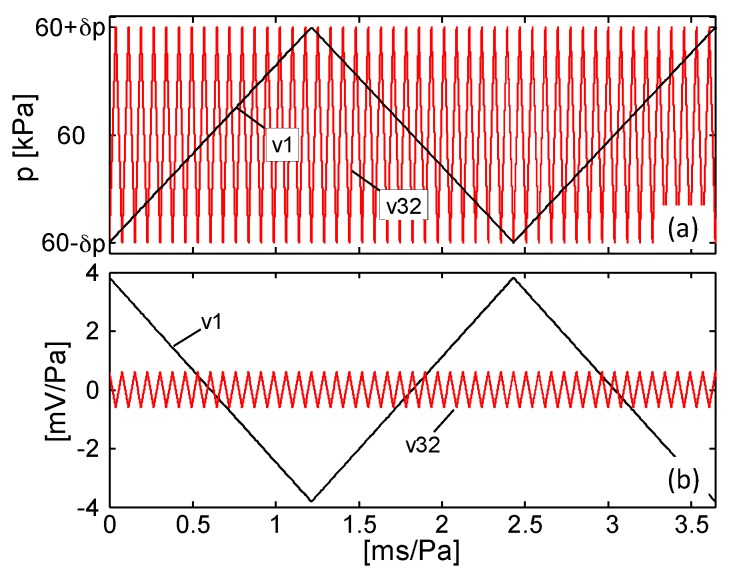
Calculated output signal (**b**) of a sensor pre-compressed at 60 kPa and subjected to a pressure variation of ±*δp* which is applied with a deformation speed of *v*_1_ or *v*_32_ (**a**).

In order to further evaluate the performance of the MLG-PDMS foam sensor, the curves in Figure 14 are used to calculate the sensitivity *S* [35] as a function of the pressure for the different applied strain rates. Firstly, each curve is fitted by a piecewise linear function, constituted by five segments defined over the pressure intervals [0, 10), [10, 20), [20, 30), [30, 50) and [50, 70] kPa. The sensitivity associated to the *j*^th^ straight-line section yields:

(4) $$\begin{array}{cccc} S_{j}=\frac{\Delta G_{\%j}}{100\cdot \Delta p_{j}} & \text{with} & j=1,2\ldots 5 & \left[{\text{kPa}}^{-1}\right] \end{array}$$

where Δ*G*_%*j*_ is the percent conductance variation over the corresponding pressure range Δ*p_j_*. The obtained results reported in Figure 18 show that *S* assumes a constant value in each operating pressure range [36]. It is observed that the value of *S*, for pressure lower than *p*_lim_ = 10 kPa, is nearly zero at the crosshead speed *v*_1_, but it assumes negative values at higher speeds due to the positive piezoresistive effect described above. As the applied pressure increases, the sensor response is characterized by a higher and higher sensitivity and *S* changes its sign at greater strain rates. In particular, the sensitivity reaches surprisingly the maximum value of ~0.23 kPa^−1^ between 50 kPa and 70 kPa, which is more than hundred times higher than the one obtained with PDMS-CNTs foams based sensors [10]. To better depict the potentiality of the new sensor developed in this work, Figure 19 shows the estimated electrical signal response of the produced MLG-PDMS foam (having the mechanical and electrical characteristics of Figure 9 and Figure 14) when, after a pre-compression of 60 kPa, the sample is subjected to a small triangular pressure oscillation of ±*δp* [Pa]. The calculation is performed considering two different pressure change rates, related to the minimum deformation speeds *v*_1_ and to the maximum one *v*_32_ used in the previous experimental tests (Figure 19a). The corresponding voltage variation Δ*V* normalized with respect to *δp* is estimated assuming that the piezoresistive sensor is supplied with a current of 100 μA. The results in Figure 19b demonstrate that for pressure variations around 1 Pa the output voltage signal is in the range of a few millivolts, and consequently it can be detected easily.

Therefore, the proposed material seems to be promising for the realization of pressure sensors in a broad range of applications. The high sensitivity in the medium-pressure regime (10–100 kPa) makes it interesting for wearable health care systems and human–machine interfacing devices. For instance, the capabilities to detect low-pressure (<10 kPa) and even subtle-pressure (1 Pa–1 kPa) variations is crucial as regards the development of ultra-sensitive e-skin [1]. 

## 4. Conclusions

In this work, we produced a lightweight pressure sensor, based on a MLGs coated polymeric foam through a cost effective technique. Firstly, a template particle leaching method is used to obtain two sets of PDMS foam (F10 and F20), differing on the stoichiometric ratio between prepolymer and curing agent (10:1 and 20:1, respectively). The different crosslinking degree affects the mechanical properties of the foams reducing the F20 elastic modulus by a factor of ~1.69 with respect to the F10 one.

Then, the produced foams are infiltrated via the drop casting of MLG-based colloidal suspensions. The effective conductivity plots of the two types of coated PDMS porous samples (MLG-F10 and MLG-F20), measured varying the weight percentage of the infiltrated MLGs show similar percolation thresholds of 0.39 wt.% and 0.38 wt.%, respectively. Therefore, due to their equivalent dc electrical behavior and the lower elastic modulus of the F20 foams the work has been focused on the final characterization of the piezoresistive behavior of the MLG-F20 material specimens infiltrated with 0.96 wt.% of MLGs. 

In particular, the performance of MLG-F20 as pressure sensor is investigated through uniaxial quasi-static compression tests at different crosshead speeds assessing the relative percentage electrical conductance variation and sensitivity as function of the applied load. The sensor response, after the mechanical stabilization procedure, follows well the applied pressure and does not show noticeable resistive drift. The tests have also demonstrated that the sensor behavior is generally affected by the strain rate. In particular, for pressures < 10 kPa, corresponding to the linear-plateau regime of the porous material, the sensor can manifest a positive piezoresistivity especially at higher crosshead speeds due to the break of MLG conductive paths. 

At higher pressures, in the near-densification regime of the foam, the conductance starts to increase rapidly; the results showed an 800% relative conductance change and an high sensitivity of 0.23 kPa^−1^ with a maximum applied pressure of 70 kPa. Further calculations have also proved that the proposed sensor, properly pre-stressed, would allow to detect pressure variations as low as 1 Pa.

## Figures and Tables

**Table 1:** Average physical characteristics of the produced PDMS foam samples, infiltrated or not with MLG suspension.

Foam Type	*ρ_PDMS_* [kg/m^3^]	*ρ_foam_* [kg/m^3^]	Φ [%]	*E_PDMS_* [MPa]	*E_foam_* [MPa]
F10	1027.8 ± 5.5	358.0 ± 13.2	65.2 ± 1.6	2.69	0.11
F20	1028.7 ± 9.0	366.8 ± 18.7	64.3 ± 1.6	0.74	0.065

**Table 2:** Amount of suspension used for PDMS foam infiltration and weight percentage of MLGs with respect to the PDMS weight for F10 and F20 foam families.

**Drop-Casted Suspension [mL]**		0.75	1	1.5	2	3	4.5
**Infiltrated MLGs [wt.%]**	**MLG-F10**	0.29	0.44	0.57	0.85	1.23	1.81
	**MLG-F20**	0.28	0.39	0.67	0.96	1.17	1.70

**Table 3:** Fitting parameters.

	*θ_c_* [wt.%]	*t*	R-Square
MLG-F10	0.39	2.35	0.982
MLG-F20	0.38	2.35	0.981

## Abbreviations

The following abbreviations are used in this manuscript:

PDMS	Polydimethylsiloxane
MLGs	Multilayer graphene nanoplatelets
DC	Direct Current
3D	Three-dimensional
CVD	Carbon vapour deposition
CNT	Carbon nanotube
PU	Polyurethane
rGO	Reduced graphene oxide
2D	Two-Dimensional
SEM	Scanning electron microscopy
AFM	Atomic force microscopies
GIC	Graphite intercalation compound
WEG	Worm-like expanded graphite
FE-SEM	Field Emission Scanning Electron Microscope
